# Age-Related Performance in Using a Fully Immersive and Automated Virtual Reality System to Assess Cognitive Function

**DOI:** 10.3389/fpsyg.2022.847590

**Published:** 2022-03-11

**Authors:** Ngiap Chuan Tan, Jie En Lim, John Carson Allen, Wei Teen Wong, Joanne Hui Min Quah, Paulpandi Muthulakshmi, Tuan Ann Teh, Soon Huat Lim, Rahul Malhotra

**Affiliations:** ^1^Duke-NUS Medical School, Singapore, Singapore; ^2^SingHealth Polyclinics, Singapore, Singapore; ^3^SingHealth Duke-NUS Family Medicine Academic Clinical Programme, Duke-NUS Medical School, Singapore, Singapore; ^4^Centre for Quantitative Medicine, Duke-NUS Medical School, Singapore, Singapore; ^5^SingHealth Polyclinics-Outram, SingHealth Polyclinics, Singapore, Singapore; ^6^Technology Development Centre, Institute of Technical Education College West, Singapore, Singapore; ^7^Centre for Ageing Research and Education, Duke-NUS Medical School, Singapore, Singapore; ^8^Health Services and Systems Research, Duke-NUS Medical School, Singapore, Singapore

**Keywords:** cognition, virtual reality, age, performance score, time

## Abstract

**Introduction:**

Cognition generally declines gradually over time due to progressive degeneration of the brain, leading to dementia and eventual loss of independent functions. The rate of regression varies among the six cognitive domains (perceptual motor, executive function, complex attention, learning and memory, social cognition and language). Current modality of cognitive assessment using neuropsychological paper-and-pencil screening tools for cognitive impairment such as the Montreal Cognitive Assessment (MoCA) has limitations and is influenced by age. Virtual reality (VR) is considered as a potential alternative tool to assess cognition. A novel, fully immersive automated VR system (Cognitive Assessment using Virtual Reality, CAVIRE) has been developed to assess the six cognitive domains. As cognition is associated with age, VR performance is postulated to vary with age using this system.

**Aims:**

This is a feasibility study to evaluate the VR performance of cognitively healthy adults aged between 35 and 74 years old, based on the performance score and completion time using the CAVIRE system.

**Methods:**

Conducted in a public primary care clinic in Singapore, 25 multi-ethnic Asian adults were recruited in each of the four age groups in years: (1) 35–44; (2) 45–54; (3) 55–64, and (4) 65–74. The eligibility criteria included a MoCA score of 26 or higher to reflect normal cognition and understanding English instructions. They completed common daily activities ranging from brushing teething to shopping, across 13 VR segments. Their performances scores and completion time were automatically computed by the CAVIRE system. These VR performance indices were compared across the four age groups using one-way ANOVA, *F*-test of the hypothesis, followed by pair-wise comparisons in the event of a significant *F*-test (*p* < 0.05).

**Results:**

One participant dropped out from Group 1. The demographic characteristics of 99 participants were similar across the 4 age groups. Overall, younger participants in Groups 1 and 2 attained higher VR performance scores and shorter completion time, compared to those in Groups 3 and 4, in all six cognitive domains (all *p* < 0.05).

**Conclusion:**

The CAVIRE VR performance scores and completion time significantly differ between the younger and older Asian participants with normal cognition. Enhancements to the system are needed to establish the age-group specific normal performance indices.

## Introduction

Dementia is prevalent globally. According to the World Health Organisation, approximately 50 million people worldwide are affected by dementia in 2019, and this figure is projected to triple to 152 million by 2050 (World Health Organisation, 2019).

Being a progressive neurodegenerative disorder, dementia involves gradual cognitive decline over time, leading to eventual loss of independent functions. The decline varies across the six cognitive domains. According to the Diagnostic and Statistical Manual of Mental Disorders (DSM-5), the six domains of cognitive function include: perceptual-motor function, executive function, complex attention, social cognition, learning and memory, and language (American Psychiatric Association, 2013). Therefore, early identification of cognitive impairment in any domain is pivotal to initiate appropriate interventions to retard its further decline (Brasure et al., 2018).

At present, the standard cognitive assessment method involves traditional neuropsychological paper-and-pencil screening tools for cognitive impairment such as the Montreal Cognitive Assessment (MoCA) and the Mini-Mental State Examination (MMSE) (Abd Razak et al., 2019). Neuropsychological tests are influenced by sociodemographic variables such as age, number of years of education, gender and cultural background (Acevedo et al., 2007). Younger age is associated with better performance among cognitively healthy participants in various neuropsychological tests, including the MoCA (Larouche et al., 2016); and the MMSE (Piccinin et al., 2013).

Neuropsychological paper-and-pencil tests have shown to be lacking in ecological validity, where the individual’s performance on the tests do not adequately predict the real-world functioning of the individual (Spooner and Pachana, 2006). On the other hand, studies have shown that virtual reality has the potential for enhanced ecological validity to provide a better assessment of the individual’s cognitive function in the real-world setting (Parsons, 2015).

In recent years, virtual reality (VR) has been deployed to assess cognition. By wearing a head-mounted device, VR enables a person to experience simulated real-life situations. VR allows the evaluation of multiple cognitive domains such as memory and executive functions. It can potentially be self-administered, requires minimal training, provides a pleasant experience and decreases the psychological distress caused by using conventional screening tools (Jin et al., 2020). Studies have demonstrated the promising applications of VR in cognition evaluation. Vallejo et al. (2017) used VR to assess cognitive processes and everyday life, including a virtual cooking scenario. Davison et al. (2017) developed a virtual parking simulator for cognitive assessment.

Age affects both cognition and VR performance. Cognition declines with age, although younger adults can be affected by Alzheimer’s Disease and other forms of dementia. Studies have revealed that generally younger individuals have better VR performance compared to those who are older (Davison et al., 2017; Ouellet et al., 2018; Plechatá et al., 2019). Sakai et al. (2018) reported significant differences in VR performance scores across age bands of 20 years, and the decline of VR performance is more obvious from the fifth decade onward. However, the results of these studies are often based on their participation in limited number of VR tasks. The study by Plechatá et al. (2019) is an example. They assessed participants’ recall of a shopping list to complete their virtual supermarket task. Overall, very little research has been done on using VR to assess cognitive function based on all six cognitive domains. Moreover, evidence is scarce on the implementation of a single VR system to assess the cognitive function of individuals across a wide span of age groups.

Therefore, the need to design a more comprehensive VR system is imperative. A novel VR system that is capable of giving automated audio-visual instructions while individuals perform tasks that cover all six cognitive domains has been developed for Asians living in urban setting (Lim et al., 2021). Known as CAVIRE (Cognitive Assessment using VIrtual REality) system, it is developed to assess the cognition of community dwelling, ambulatory, older multi-ethnic Asians living in densely populated housing estates.

In this paper, we have recruited participants from different age groups, ranging from 35 years old to 74 years old, and assessed their cognitive function using CAVIRE based on the six cognitive domains. By assessing the performance indicators for each of the six cognitive domains, we would like to establish the system-specific normogram of VR performance for adults across different age groups. Such a normogram is crucial to identify adults with abnormal cognition based on their VR performance in the context of their age group.

Cognitively healthy younger adults are postulated to achieve better performance scores and shorter completion time compared to cognitively healthy older people. Understanding the differential VR performance across the different age groups and cognitive domains will enhance the utility potential of CAVIRE to assess cognition in normal aging.

## Aims

This is a feasibility study aimed to assess the performance of cognitively healthy Asian adults aged between 35 and 74 years old to complete tasks in a fully immersive and automated VR system. Their performance was evaluated using a score matrix on the correct attempts and the time taken to complete the tasks across thirteen virtual segments.

## Materials and Methods

This manuscript presents the results from a sub-analysis of a published study protocol on the CAVIRE system (Lim et al., 2021).

### The Cognitive Assessment Using Virtual Reality System

The system includes a pre-assessment tutorial session, thirteen virtual segments to assess cognitive function, and an automated scoring system.

During the tutorial session, participants put on a head-mounted display device (HMD), namely HTC VIVE Pro, and are inducted to the VR environment by using head movements and hand movements respectively. The hand movements are recorded by the Leap Motion controller. It is a small USB peripheral device mounted onto the HMD. Using two monochromatic infra-red (IR) cameras and three IR LEDs, the device observes a roughly hemispherical area, to a distance of about one meter. The LEDs generate pattern-less IR light and the cameras generate almost 200 frames per second of reflected data. This is then sent through a USB cable to the computer, where it is analyzed by the Leap Motion software using complex algorithms. The 3D position data of the hand movements is synthesized by comparing the 2D frames generated by the two cameras.

The Voice Recognition software utilizes built-in microphone from Windows PC for the voice input. The Application Programming Interface (API) is integrated within the system. It will automatically detect the participant’s speech without recording or active speech extraction. However, for the purposes for our study, we use an external USB microphone. The Voice Recognition software is customized to detect the participant’s correct pronunciation of multiple fruit names in English with a Graphic User Interface (GUI) feedback indication. They are integrated and run in parallel with other Unity3D Applications of the CAVIRE software.

Once participants indicate readiness to the researcher, they proceed to begin with the VR segments. Each segment features common day-to-day activities to assess specific domains of cognitive function. These activities are contextualized to the local Singapore setting and would be familiar to both the young and old participants. Participants perform these virtual tasks via hand gestures and head movements which are detected by motion sensors. Their speech is assessed using the Voice Recognition technology embedded in the system. The automated voice and visual instructions in English guide the participants to complete the tasks.

The participants perform the VR tasks across the following thirteen segments:

(1) Brushing and rinsing teeth.
(2) Preparing peanut butter bread for breakfast.
(3) Identifying pictures of important persons in the newspaper.
(4) Watching television, while listening to the weather forecast regarding impending rain on the radio.
(5) Naming the fruits in a shopping list and remembering the fruits.
(6) Choosing the appropriate clothing to go for grocery shopping.
(7) Remembering to pick up the umbrella, before opening and locking the door.
(8) Taking the lift to level 1 in an apartment block by pressing the correct buttons.
(9) Looking to the left and right, and waiting for green pedestrian light, before crossing the street.
(10) Remembering and choosing the stipulated stall, i.e., the one which sells fruits.
(11) Picking the correct fruits based on recall from the shopping list.
(12) Calculating and paying the correct sum of money for all the fruits.
(13) Selecting the appropriate emotion, with regards to scenes of a birthday party and car accident respectively.

**FIGURE 1:**
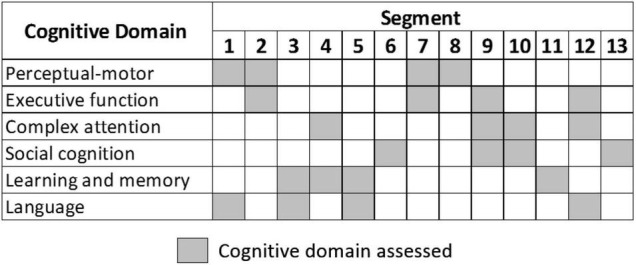
Cognitive domain assessed in each segment.

With regards to Figure 1, these thirteen segments cover the six cognitive domains, as defined by the 5th edition of the Diagnostic and Statistical Manual of Mental Disorders (DSM-5) (American Psychiatric Association, 2013). Four segments (6, 8, 11, and 13) assess a single cognitive domain, while the tasks in the remaining nine segments evaluate two or more cognitive domains. For a balanced evaluation framework, each cognitive domain is assessed over four different segments. The system was pilot-tested internally with young and old volunteers and was further enhanced over three revisions. The maximum amount of time needed to complete the VR assessment across the thirteen segments is approximately 12 min.

The CAVIRE system features an automated scoring system to assess each task performance. The scores are computed automatically for each segment and for the entire VR assessment. The scoring matrix comprises (i) the proportion of tasks performed correctly; (ii) number of attempts needed to complete each task and (iii) completion of tasks within the stipulated time. The matrix is illustrated in “Appendix Table A1 – Scoring algorithm of the VR assessment.”

### Study Site

A public primary care clinic (polyclinic) in the southern region of Singapore was determined as the study site. This polyclinic provides comprehensive primary healthcare services to an estimated population of 18,960 residents of varying Asian ethnicity in the Outram estate, of which 24.7% were aged 65 years and above in 2019 (Department of Statistics et al., 2019). About 80% of its patient population originated from other estates in Singapore, as it provides accessible healthcare services to employees working in the central business district in the proximity.

### Study Population

Participants included those who sought medical consultation at the polyclinic as well as visitors and accompanying persons of patients at the polyclinic if they satisfied the eligibility criteria: (1) aged between 35 and 74 years old, and (2) understood English (the medium of instruction in CAVIRE, and (3) willing to complete the questionnaires and the CAVIRE VR assessment, and (4) MoCA score of 26 or more.

Individuals with any of the following were excluded: pre-existing diagnosis of cognitive impairment or dementia as documented in their electronic medical record; any disability which rendered them incapable of providing written informed consent; neurological deficits that might affect vision, hearing, speech or motor skills; or known motion sickness or epilepsy.

### Sample Size

A total of 25 participants were recruited in each 10-year age group: (1) 35–44 years old; (2) 45–54 years old; (3) 55–64 years old; (4) 65–74 years old. As this is a feasibility study, sample size calculation is not necessary, but sample size justifications need to be provided (Billingham et al., 2013). The Modified Wald method was used to calculate the confidence interval of a proportion. Using a one-sided 95% confidence interval, based on a proportion of 23 out of 25 participants in a particular age group, at least 77% of participants in that age group who undertake the CAVIRE assessment in the future would be able to complete it. For the purposes of this feasibility study, a 90% completion rate, which equates to 23 out of 25 participants in each age group, would be considered adequate. Thus, VR performance of 100 cognitively healthy participants, 25 in each of four different age groups, were enrolled.

### Recruitment and Procedure During the Study Administration

Prior to the start of the study, the Research Assistant (RA) was trained by physicians and Advanced Practice Nurse on the recruitment procedure and the administration of the MoCA test. During the study implementation, the RA screened the eligibility of potential participants at the waiting area of the clinic, or via internal referral from study investigators. The RA explained the study protocol, obtained written informed consent, and confirmed the participants’ eligibility criteria from their electronic medical records before administering the MoCA.

Cognitively healthy participants, i.e., with a MoCA score of 26 or higher were enrolled. While scoring the MoCA, an additional point correction was accorded to those with ≤10 years of education (Ng et al., 2013). Those who attained a MoCA score of less than 26 were excluded and referred for further clinical assessment at the polyclinic.

The Pre-VR questionnaire gathered the participants’ demographic data (age, gender, ethnicity, number of years of education) and scores from the following validated cognitive and functional status assessments: (1) Abbreviated Mental Test (AMT) and (2) Mini-Mental State Examination (MMSE).

Next, the participants were briefed on the VR procedure and equipment. With the help of the RA, the participants sat on a chair and put on the VR head-mounted device. The participants were then introduced to a tutorial session. This tutorial session allowed all participants, regardless of age, to familiarize themselves and feel comfortable in using their head and hand movements in the VR environment. Once the participants were ready, they proceeded to complete the 13 segments of the VR assessment. The scores and the time taken to complete each segment were automatically computed in the CAVIRE system and were aggregated for their overall VR performance.

### Outcome Measurements

The CAVIRE performance-indices were computed according to the matrix for each cognitive domain in the four age groups: (1) 35–44 years old; (2) 45–54 years old; (3) 55–64 years old; (4) 65–74 years old. The aggregated indices are comprised of both the VR performance scores and completion time.

### Data Management and Monitoring

The data from the questionnaires were transcribed into Redcap, a secure research database, and audited by a data management officer in the institution for errors. The VR data from the CAVIRE system was exported to the same database and merged with the audited questionnaire data. The anonymized combined data were handed over to data analysts in the study team. Participants who complained of headache, nausea or giddiness during the VR assessment were advised to stop the procedure and considered as dropouts.

### Statistical Analysis

Potential confounders were compared among the four age groups. The potential confounders include gender (male, female), ethnicity (Chinese, non-Chinese), education level (up to secondary, post-secondary/tertiary), and housing (public, private) as a surrogate for socio-economic status. As no significant difference among the four age groups for any confounder was found, the VR performance indices (VR performance score and VR completion time) for the six cognitive domains were compared across the age groups. This was done using a one-way analysis of variance (ANOVA) *F*-test of the hypothesis H_0_: μ_1_ = μ_2_ = μ_3_ = μ_4_ (all means equal) vs. H_1_: μ_*i*_ ≠μ_*j*_ for at least one *i* ≠ *j* (*i* = *j* = 1, 2, 3, 4) (at least two means different). For the rejection of H_0_, an *F*-test *p*-value of *p* < 0.05 was considered statistically significant. This is followed by pair-wise comparisons among the four age groups for the VR performance score and the VR completion time respectively. In addition, a linear trend across the four age groups was tested using a contrast. Based on linearized Q-Q plots, the normality of residuals was assessed visually and found to be tenable. For this study, all statistical analyses were done by utilizing the software SAS v9.4.

## Results

This segment of the study commenced recruitment in October 2020 and was completed by January 2021. Only one participant from the 35 to 44 years age group failed to complete the study due to apprehension during the administration of the MoCA questionnaire, constituting a dropout rate of 1%. No participant experienced any adverse effect while performing the VR tasks.

Demographic characteristics of the remaining 99 participants are presented in Table 1. The characteristics are similar across the four age groups, labeled as Group 1 for 35–44 years old; Group 2 for 45–54 years old; Group 3 for 55–64 years old and Group 4 for 65–74 years old.

**TABLE 1:** Demographic characteristic frequency counts (%) and comparison among age groups.

Demographic characteristic		Overall (*n* = 99)	Age group (years) Count (%)				
			35–44 (*n* = 24)	45–54 (*n* = 25)	55–64 (*n* = 25)	65–74 (*n* = 25)	*p*-value
Gender	Male	44 (44.4)	12 (27.3)	11 (25.0)	12 (27.3)	9 (20.5)	0.765
	Female	55 (55.6)	12 (21.8)	14 (25.5)	13 (23.6)	16 (29.1)	
Ethnicity	Chinese	77 (77.8)	19 (24.7)	16 (20.8)	21 (27.3)	21 (27.3)	0.273
	Non-Chinese	22 (22.2)	5 (22.7)	9 (40.9)	4 (18.2)	4 (18.2)	
Education	Up to Secondary	28 (28.3)	2 (7.1)	8 (28.6)	8 (28.6)	10 (35.7)	0.081
	Post-Secondary/Tertiary	71 (71.7)	22 (31.0)	17 (23.9)	17 (23.9)	15 (21.1)	
Socio- economic status	Public housing	66 (66.7)	17 (25.8)	16 (24.2)	19 (28.8)	14 (21.2)	0.470
	Private housing	33 (33.3)	7 (21.2)	9 (27.3)	6 (18.2)	11 (33.3)	

**FIGURE 2:**
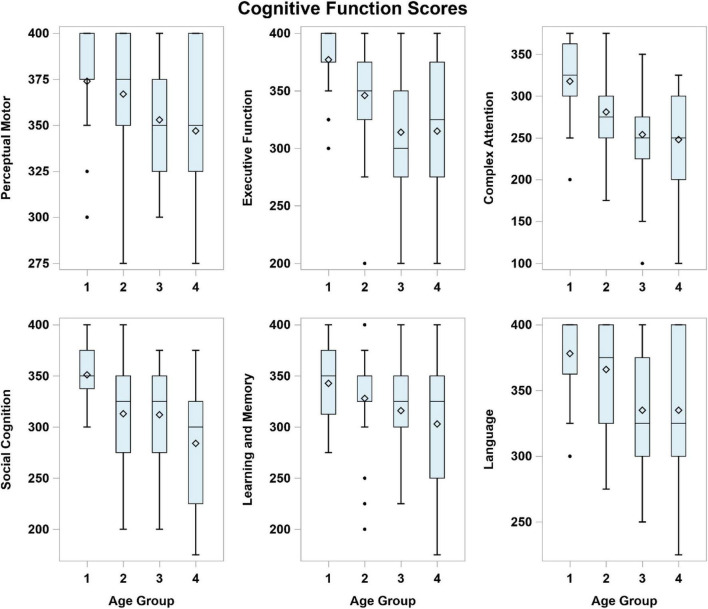
Virtual reality performance scores in each of the six cognitive domains by age group. Age Group (years): 1 = 35–44, 2 = 45–54, 3 = 55–64, 4 = 65–74.

Figure 2 shows the VR performance scores in the six cognitive domains by the four age groups. The performance score of each cognitive domain was computed based on the aggregated scores of the respective segments stipulated in the matrix (Figure 2).

Table 2 summarizes the mean (SD) performance scores for each age group and the inter-age group mean score differences. Overall, younger participants achieved higher scores compared to the older participants in each cognitive domain (all *p* < 0.05, Table 2). The inter-age group VR performance scores are discriminant in all six cognitive domains between the Groups (1) and (4); and discriminant in all cognitive domains except for “Learning and Memory” between Groups (1) and (3). Performance scores show no significant difference among the older participants in Groups (3) and (4) across the domains except for “Social Cognition.”

**TABLE 2:** Virtual reality mean (SD) performance score by age group with differences and pair-wise comparisons.

Cognitive domain	Age group Mean (SD) score				*P*-values: *F*-test (Linear trend)	Pair-wise differences between age groups 95% confidence interval on difference and *p*-value					
	(1) 35–44 years (*n* = 24)	(2) 45–54 years (*n* = 25)	(3) 55–64 years (*n* = 25)	(4) 65–74 years (*n* = 25)		(1) vs. (2)	(1) vs. (3)	(1) vs. (4)	(2) vs. (3)	(2) vs. (4)	(3) vs. (4)
Perceptual Motor	374.0 (29.9)	367.0 (29.9)	353.0 (29.2)	347.0 (45.8)	0.0386 (0.0044)	6.96 (–13.5, 27.4) 0.501	21.0 (0.50, 41.4) 0.045*	27.0 (6.50, 47.4) 0.010**	14.0 (–6.25, 34.2) 0.173	20.0 (–0.25, 40.2) 0.053	6.00 (–14.2, 26.2) 0.558
Executive Function	377.1 (32.9)	346.0 (44.3)	314.0 (52.1)	315.0 (61.2)	<0.0001 (<0.0001)	31.1 (3.34, 58.8) 0.029*	63.1 (35.3, 90.8) <0.001***	62.1 (34.3, 89.8) <0.001***	32.0 (4.55, 59.5) 0.023*	31.0 (3.55, 58.5) 0.027*	–1.00 (–28.5, 26.5) 0.943
Complex attention	317.7 (46.9)	281.0 (44.1)	254.0 (55.8)	248.0 (66.9)	<0.0001 (<0.0001)	36.7 (5.95, 67.5) 0.020*	63.7 (33.0, 94.5) <0.001***	69.7 (39.0, 100.5) < 0.001***	27.0 (-3.44, 57.4) 0.082	33.0 (2.56, 63.4) 0.034*	6.00 (−24.4, 36.4) 0.697
Social cognition	351.0 (27.1)	313.0 (53.6)	312.0 (44.6)	284.0 (59.5)	<0.0001 (<0.0001)	38.0 (10.8, 65.2) 0.007**	39.0 (11.8, 66.2) 0.005**	67.0 (39.8, 94.2) <0.001***	1.00 (–25.9, 27.9) 0.941	29.0 (2.09, 55.9) 0.035*	28.0 (1.09, 54.9) 0.042*
Learning and memory	342.7 (40.0)	328.0 (44.1)	316.0 (46.1)	303.0 (67.4)	0.0474 (0.0051)	14.7 (–14.0, 43.4) 0.312	26.7 (–2.03, 55.4) 0.068	39.7 (11.0, 68.4) 0.007**	12.0 (–16.4, 40.4) 0.404	25.0 (–3.44, 53.4) 0.084	13.0 (–15.4, 41.4) 0.367
Language	378.1 (34.0)	366.0 (42.6)	335.0 (49.0)	335.0 (59.5)	0.0020 (0.0003)	12.1 –14.7, 39.0) 0.372	43.1 (16.3, 70.0) 0.002**	43.1 (16.3, 70.0) 0.002**	31.0 (4.43, 57.6) 0.023*	31.0 (4.43, 57.6) 0.023*	0.00 (–26.6, 26.6) 1.000

*, **, *** Statistically significant at p < 0.05, p < 0.01, p < 0.001.

For specific cognitive domains (Table 2), significant differences are noted in sequential age groups from (1) to (3) in “Executive Function”. In the “Perceptual Motor” domain, the performance scores differ between the youngest participants in Group (1) and older participants in Groups (3) and (4). In “Complex Attention” and “Social Cognition” domains, differential VR performances were recorded between younger participants in Group 1 and those in Groups (2), (3), and (4). In “Learning and Memory,” the only significant difference is between the extreme age groups (1) and (4). In “Language,” significant differences in performance scores are noted between younger and older participants [i.e., Groups (1) vs. (3), (1) vs. (4), (2) vs. (3), and (2) vs. (4)].

**FIGURE 3:**
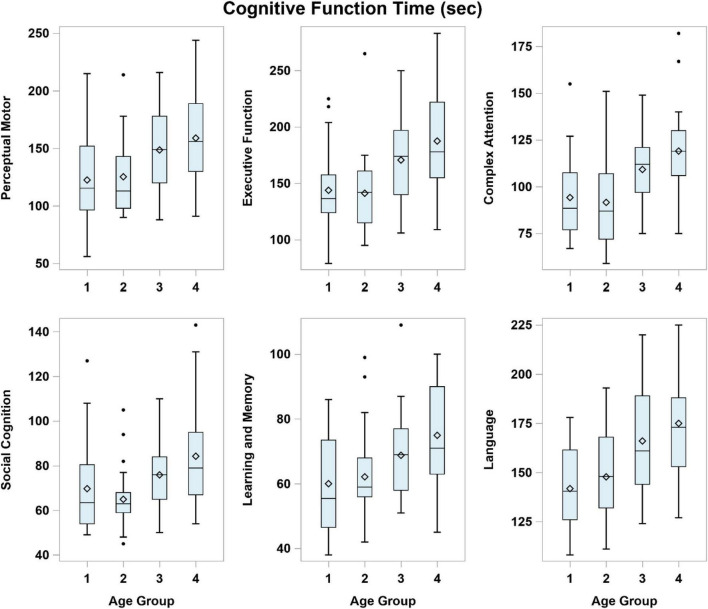
Time to complete the virtual reality tasks in each cognitive domain across the age groups. Age Group (years): 1 = 35–44, 2 = 45–54, 3 = 55–64, 4 = 65–74.

Figure 3 summarizes the time to complete the VR tasks in each cognitive domain. Overall, younger participants took a shorter time to complete the VR tasks compared to those in the older age groups regardless of the cognitive domain (all *p* < 0.01, Table 3).

**TABLE 3:** Virtual reality mean (SD) completion time by age group with differences and pair-wise comparisons.

Cognitive domain	Age group Mean (*SD*) time taken (seconds)				*P*-values: *F*-test (Linear trend)	Pair-wise differences between age groups 95% confidence interval on difference and *p*-value					
	(1) 35–44 years (*n* = 24)	(2) 45–54 years (*n* = 25)	(3) 55–64 years (*n* = 25)	(4) 65–74 years (*n* = 25)		(1) vs. (2)	(1) vs. (3)	(1) vs. (4)	(2) vs. (3)	(2) vs. (4)	(3) vs. (4)
*Perceptual motor*	122.5 (37.0)	125.4 (32.5)	148.6 (36.1)	159.0 (38.8)	0.0009 (<0.0001)	–2.82 (–23.3, 17.7) 0.786	–26.1 (–46.6, –5.58) 0.013*	–36.5 (–57.0, –15.9) <0.001***	–23.3 (–43.6, –2.97) 0.025*	–33.6 (–53.9, –13.3) 0.001***	–10.4 (–30.7, 9.95) 0.314
*Executive function*	143.9 (36.9)	141.2 (34.9)	170.7 (37.2)	187.5 (47.0)	<0.0001 (<0.0001)	2.64 (–19.7, 24.9) 0.815	–26.8 (–49.1, –4.51) 0.019*	–43.6 (–65.9, –21.4) <0.001***	–29.4 (–51.5, –7.38) 0.010**	–46.3 (–68.3, –24.2) <0.001***	–16.8 (–38.9, 5.22) 0.133
*Complex attention*	94.3 (20.6)	91.6 (23.7)	109.2 (18.8)	119.1 (27.0)	<0.0001 (<0.0001)	2.61 (–10.3, 15.5) 0.689	–15.0 (–27.9, –2.07) 0.024*	–24.8 (–37.8, –11.9) <0.001***	–17.6 (–30.4, –4.81) 0.008**	–27.4 (–40.2, –14.7) <0.001***	–9.84 (–22.6, 2.95) 0.130
*Social cognition*	69.8 (20.3)	65.0 (13.5)	75.9 (16.1)	84.3 (24.1)	0.0036 (0.0019)	4.75 (–5.98, 15.5) 0.382	-6.17 (–16.9, 4.56) 0.257	–14.5 (–25.3, –3.80) 0.009**	–10.9 (–21.5, –0.30) 0.044*	–19.3 (–29.9, –8.66) < 0.001***	–8.36 (–19.0, 2.26) 0.1215
*Learning and memory*	60.0 (14.92)	62.2 (13.78)	68.8 (51.00)	75.0 (45.0)	0.0017 (0.0002)	–2.12 (–10.3, 6.07) 0.609	–8.76 (–16.9, –0.57) 0.036*	–14.9 (–23.1, –6.73) <0.001***	–6.64 (–14.7, 1.47) 0.107	–12.8 (–20.9, –4.69) 0.002**	–6.16 (–14.3, 1.95) 0.135
*Language*	141.8 (20.0)	147.8 (20.0)	166.0 (28.6)	175.0 (29.2)	<0.0001 (<0.0001)	–5.97 (–20.6, 8.62) 0.419	–24.2 (–38.8, –9.62) 0.001***	–33.2 (–47.8, –18.6) <0.001***	–18.2 (–32.7, –3.80) 0.014**	–27.2 (–41.6, –12.8) <0.001***	–8.96 (–23.4, 5.48) 0.221

*, **, *** Statistically significant at p < 0.05, p < 0.01, p < 0.001.

Table 3 summarizes the mean (SD) completion time for each age group and the inter-age group mean score differences. The completion time is discriminant in all six cognitive domains between the younger participants in Groups (1) and (2) compared to older participants in Group (4) respectively. Significant differences in completion time are noted between Groups (1) and (3) except for “Social Cognition” and between Groups (2) and (3) except for “Learning and Memory”. Completion time is similar between the younger participants in Groups (1) and (2) across all cognitive domains (Table 3).

In this study, all statistical analysis methods were parametric, as well as assuming normal error distributions. The assumption of normality of the ANOVA residuals was evaluated, and subsequently found to be tenable. All comparisons were done using ANOVA *F*-tests, followed by *t*-tests for pair-wise comparisons among the four age groups.

## Discussion

Virtual reality is increasingly applied in cognitive assessment for neurocognitive disorders, conferring merits and advantages over the paper-and-pencil methods (Jin et al., 2020). The results show differentiation of the CAVIRE-based VR performance indices between the younger and older cognitively healthy adults across six cognitive domains. This study distinguishes itself from most VR studies that assessed cognitive function on older adults. Bottiroli et al. (2017) validated a VR platform using serious games, known as Smart Aging, for assessing cognitive functions in normal aging. Their participants were largely Italians aged 50 years and older, whose memory, executive functions, working memory, visual spatial elaboration, language, and orientation were assessed. As for the current study, it focused on the VR performance of multi-ethnic Asian adults to complete activities of daily living in an urban environment, which could better reflect the impact of cognition on their life.

The findings in this study show that cognitively healthy younger Asian adults tended to attain higher performance scores and completed the VR within shorter time, compared to their older counterparts. The differentiation cuts across all six domains between the age groups of 35–44 and 65–74. Kinesthesia, the conscious awareness of limb position or limb motion, can influence the participant’s VR performance. Fry et al. (2003) reported age-related changes in upper extremity kinesthesia. According to their study, the younger participants demonstrated significantly lower thresholds of kinesthetic acuity and less error on the kinesthetic memory test compared with those who were older. Thus, the time-based VR performance provides another dimension to cognitive assessments, especially those involving executive function based on the brain-upper limbs axis.

Preparing a toast exemplifies such assessment of executive function, which is incorporated in segment 2 of the CAVIRE system. Such perspectives cannot be objectively evaluated via self-reporting in questionnaire-based assessments. The effect of kinesthesia in executive function alludes to the need to establish age-normalized performance indices specific to both the VR software and to the target population.

For executive function, significant declines in performance scores were noted from the 35–44 years to the 55–64 years age groups (Table 2 and Figure 2). A significant increase in the length of time to complete four tasks in this domain was noted between the 45–54 and 55–64 years age groups (Table 3 and Figure 3). Performing tasks such as preparing a toast, bringing along an umbrella in the context of a rainy day, crossing a road with signal from traffic lights and settling payment by selecting the correct currency notes are reflective of day-to-day executive functions. Such activities of daily living are difficult to assess without direct and objective observations in a clinical setting. Executing such simulated tasks requires reasoning and problem solving. This finding is compatible to the decline in executive function performance using VR from the fifth decade onward, as reported by Sakai et al. (2018). The results of this study suggest that while the performance score decreases markedly in participants aged around mid-50s, the VR performance among the older age groups seems to be stable from 55 to 74 years of age (Table 2).

The VR performance scores for social cognition decreased significantly between the younger age groups and between the older age groups (Table 2). However, the VR completion time shows a distinct increase with age, shorter for the younger (up to 54 years) and longer for the older age groups (55 years and older). The selection of clothing, stall and emotions in the appropriate context are the main tasks in this cognitive domain. Broadly, social cognition includes processes such as social interactions, which are deemed appropriate in the local social context. It represents a complex integration of cognitive functions which are organized to deal with complex processing demands. The conventional paper-and-pencil cognitive assessments have limitations in evaluating social cognition. The study results suggest the potential of VR to screen for deficits in social cognition based on the performance score and completion time in people of different age groups.

Complex attention refers to a person’s ability to maintain information in their mind for a short time and to manipulate that information. As the demands on attention increase, performance slows down and information is less able to be retained. Segment 4 is one of the four settings to provide insight on complex attention: a participant watches a television program while listening to the weather forecast on impending rain over the radio. The results show significant decline in performance score between the 35–44 years and 45–54 years age groups, and longer completion time between the 45–54 years and 55–64 years age groups. Complex attention seems to be similar for the older participants aged 55 years and older.

Language is based on the understanding of the English instructions to perform the stipulated task. Naming the fruits is a good example to demonstrate this cognitive domain. Both the performance score and completion time show significant difference between the 45–54 and 55–64 years age groups. Incorporating voice recognition technology in the CAVIRE software provides objective evaluation of a participant’s articulation of the fruits’ names. Such integration of voice recognition and VR technologies to assess cognition is novel and likely synergistic. The combined applications have to be further evaluated when culturally adapted editions of CAVIRE in other languages are developed to cater to the multi-lingual Asian adults.

Virtual reality completion time is shown to distinguish the perceptual motor domain between the 45–54 and 55–64 years age groups. Activating the lift button to get to the correct floor is one of the four segments to assess perpetual motor function. The completion time is a reflection of the processing speed to carry out the task. Younger participants seem to accomplish such activity more rapidly than their older counterparts. Even the completion time varies across the younger participants in the 35–44 and 45–54 years age groups, although the differences are not statistically significant (Figure 3 and Table 3). Zhang et al. (2001) had also revealed that the VR completion time varied in the control group of younger healthy participants in a trial to compare with the VR performance of their counterparts with brain injury. Nevertheless, no difference is noted in their performance scores across the age groups. This calls for a review in the matrix score board to improve its discrimination of perceptual motor function.

In summary, the VR performances of Asian adults varied with age in using the CAVIRE system, with variable discrimination among the different cognitive domains. Nonetheless, users of this system should be cognizant of the differences in the performance scores and completion time between the younger and older participants, with the most significant demarcation around the age of 55 years old. Further refinements to the VR segments and scoring matrix are needed to reduce the wide dispersion of the scores and completion time.

## Strengths and Limitations

The study shows the unprecedented ability of VR technology (CAVIRE) to discriminate the six cognitive domains according to the performance score and completion time in adults aged between 35 and 74 years old with healthy cognition (based on the MoCA). The CAVIRE system also shows significant differences in VR performance across the age groups, regardless of the cognitive domains. The voice recognition incorporated in the VR system is novel and allows objective assessment of language. Older participants were receptive of using the headset and completed the VR tasks without experiencing any adverse effects. No participants dropped out from the study due to failure to use the VR equipment. The embedded automated scoring system standardizes the appraisal of the VR tasks and measures the associated completion time. The results of this study complement that of a previous publication on CAVIRE, where the entire completion time for CAVIRE was significantly shorter compared to that of MoCA (Wong et al., 2021). These results show that CAVIRE is feasible to be used for cognitive assessment in the six cognitive domains for individuals across different age groups.

Nevertheless, the study has its limitations. The limited number of participants in each age group restricts the generalizability of this study to the general population. Cognitively healthy participants were identified using only a single MoCA cut-off score of 26. According to a multi-ethnic meta-analysis, a MoCA cut-off of 26 may not be suitable (Carson et al., 2017). However, it is important to note that the meta-analysis was based on different ethnic populations, which also included non-Asians. This current study on Asian individuals is contextualized to Singapore, in which a MoCA cut-off score of 26 is widely used (Ng et al., 2013).

Although no significant difference was observed in the basic demographic characteristics of the participants across the age groups, including education level, it was noted that a higher proportion of the younger participants (35–44 years old) received post-secondary/tertiary education. Nevertheless, it is possible that the effect of education was not accentuated, since the tasks in the CAVIRE assessment consist of common activities of daily living. Other studies on VR have also shown that the effect of education is not significant (Oliveira et al., 2018). In the current study, participants were stratified according to different age groups, but due to the limited sample size, stratification according to education level was not possible. Potential confounders such as intellectual capacity, occupations and intra-age group variations were also not addressed in this study.

The performance scores for each cognitive domain were aggregated based on four different segments to mitigate execution variations and functional assessment in a single segment. The segments will require review and refinement to better differentiate the cognitive functions. An adequately powered randomized controlled trial using an enhanced edition of CAVIRE system will be developed to attest its discrimination in cognitive assessment across the different age groups, compared to conventional paper-and-pencil-based assessments.

## Conclusion

The study shows that the VR performance scores and completion time of using the fully immersive and automated CAVIRE system significantly differ between the younger and older Asian adults with healthy cognition. The system has shown potential to assess adults across the six cognitive domains although its degree of discrimination varies between domains. Enhancements to the VR segments and matrix score are needed to establish the age group-specific normal performance indices.

## Data Availability Statement

The original contributions presented in the study are included in the article, further inquiries can be directed to the corresponding author.

## Ethics Statement

The studies involving human participants were reviewed and approved by SingHealth Centralised Institutional Review Board, Singapore. The patients/participants provided their written informed consent to participate in this study.

## Author Contributions

JL, WW, JQ, and NT designed the VR performance tasks and score matrix and the study protocol. RM reviewed the face validity of the 13 segments for cognitive assessment. TT and SL collaborated with FXMedia Internet Pte Ltd, the industrial collaborator to develop the VR system, and including the voice recognition component. WW and NT secured the funding. PM and JL conducted the study and collated and audited the data. JA analyzed the data. NT, JA, JL, RM, WW, and JQ interpreted the results. JL and NT drafted the manuscript. All the authors reviewed, critiqued, and revised the draft, finalized and approved before submitting the manuscript to the journal.

## Conflict of Interest

The authors declare that the research was conducted in the absence of any commercial or financial relationships that could be construed as a potential conflict of interest.

## Publisher’s Note

All claims expressed in this article are solely those of the authors and do not necessarily represent those of their affiliated organizations, or those of the publisher, the editors and the reviewers. Any product that may be evaluated in this article, or claim that may be made by its manufacturer, is not guaranteed or endorsed by the publisher.

## Appendix

**TABLE A1:** Scoring algorithm of the VR assessment.

Segment	Task	Score					Remarks	Cognitive Domain(s) assessed	Given time (sec)
		0	25	50	75	100			
1	Following step-by-step instructions: (1) Squeeze toothpaste on toothbrush (2) Brush teeth (3) Rinse mouth	No attempt	Attempts, but unable to complete any Tasks	Complete 1 Task	Complete 2 Tasks	Complete all 3 Tasks	NIL	Perceptual motor Language	90
2	Preparing peanut butter bread without specific instructions: (1) Open peanut butter jar (2) Take peanut butter using knife (3) Spread peanut butter on bread	No attempt	Attempts, but unable to complete any Tasks	Complete 1 Task	Complete 2 Tasks	Complete all 3 Tasks	NIL	Perceptual-motor Executive function	90
3	Identify 3 images of important persons in the newspaper: (1) Lee Kuan Yew (2) Halimah Yacob (3) Goh Chok Tong	No attempt	Attempts, but unable to identify any images correctly	Identify 1 image correctly	Identify 2 images correctly	Identify all 3 images correctly	NIL	Learning and memory Language	30
4	(1) Remember to take umbrella before leaving the house later >>> Television acts as a distractor >>> Radio gives weather forecast of rain	NIL	NIL	NIL	NIL	NIL	This Task will be scored in Segment 7(b)	Complex attention Learning and memory	NA
5	(1) Name the five fruits by reading out aloud: Apple, Banana, Watermelon, Mango, Durian (2) Remember the five fruits	No attempt	Attempts, but unable to name any fruits correctly	Name 1–2 fruits correctly	Name 3–4 fruits correctly	Name 5 fruits correctly	Task #2 will be scored in Segment 11	Learning and memory Language	50
6	(1) Choose appropriate clothing (female/male) to go out for shopping	No attempt	Attempts, but unable to choose the correct clothing	Choose the correct clothing in 2 attempts or more	Choose the correct clothing in 1 attempt, with a time of 15 s or more	Choose the correct clothing in 1 attempt, with a time of less than 15 s	NIL	Social cognition	30
7(a)	(1) Open the door (2) Select the correct item to lock the door (3) Lock the door	No attempt	Attempts, but unable to complete any Tasks	Complete 1 Task	Complete 2 Tasks	Complete all 3 Tasks	NIL	Perceptual-motor Executive function	60
7(b)	Remember to take umbrella before leaving the house >>> Hint given at two time points: (i) before locking the door (ii) after locking the door	Does not remember to take the umbrella at all	Remember to take the umbrella after closing the door outside the house (hint is given for the second time)	Remember to take the umbrella before closing the door outside the house (hint is given for the first time)	Remember to take the umbrella after opening the door inside the house (before any hints are given)	Remember to take the umbrella before opening the door inside the house (before any hints are given)	Continued from Segment 4	Continued from Segment 4	NA
8	(1) Press button to go down (outside lift) (2) Press button for Level 1 (inside lift)	No attempt	Attempts, but unable to complete any Tasks	Complete both Tasks in 2 attempts or more respectively	Complete 1 Task in 1 attempt and Complete the other Task in 2 attempts or more	Complete Task #1 in 1 attempt and Complete Task #2 in 1 attempt	NIL	Perceptual-motor	20
9	(1) Press “Start” to cross after traffic light turns green (2) Looks to the left before crossing (3) Looks to the right before crossing	No attempt	Attempts, but unable to complete any Tasks	Complete 1 Task	Complete 2 Tasks	Complete all 3 Tasks	NIL	Executive function Complex attention Social cognition	60
10	Choose the correct store: i.e., clothes, electrical appliances, vegetables, fruits	No attempt	Attempts, but unable to choose the correct store	Choose the correct store in 2 attempts or more	Choose the correct store in 1 attempt, with a total time of 15 s or more	Choose the correct store in 1 attempt, with a total time of less than 15 s	NIL	Complex attention Social cognition	40
11	Choose the five fruits based on the previous shopping list: i.e., Apple, Banana, Watermelon, Mango, Durian	No attempt	Attempts, but unable to choose any fruits correctly	Choose three fruits or less correctly	Choose four fruits correctly	Choose five fruits correctly	Continued from Segment 5	Learning and memory	50
12	Following step-by-step instructions: (1) Calculate total price of the five fruits (2) Pay exact amount of money	No attempt	Attempts, but unable to complete any Tasks	Complete both Tasks in 2 attempts or more respectively	Complete 1 Task in 1 attempt, and Complete the other Task in 2 attempts or more	Complete Task #1 in 1 attempt, and Complete Task #2 in 1 attempt	NIL	Executive function Complex attention Language	90
13	Choose the correct emotion with regards to the scene: (1) Birthday party (2) Car accident	No attempt	Attempts, but unable to complete any Tasks	Complete both Tasks in 2 attempts or more respectively	Complete 1 Task in 1 attempt, and Complete the other Task in 2 attempts or more	Complete Task #1 in 1 attempt, and Complete Task #2 in 1 attempt	NIL	Social cognition	30
